# Emerging role of SWI/SNF complex deficiency as a target of immune checkpoint blockade in human cancers

**DOI:** 10.1038/s41389-020-00296-6

**Published:** 2021-01-08

**Authors:** Min Zhou, Jianlong Yuan, Yaqi Deng, Xianqun Fan, Jianfeng Shen

**Affiliations:** 1 Department of Ophthalmology, Ninth People’s Hospital, Shanghai JiaoTong University School of Medicine, Shanghai, 200025 China; 2 Shanghai Key Laboratory of Orbital Diseases and Ocular Oncology, Shanghai, 200025 China

**Keywords:** Tumour biomarkers, Tumour immunology

## Abstract

Mammalian SWI/SNF complex is a key chromatin remodeler that reshapes nucleosomes and regulates DNA accessibility. Mutations in SWI/SNF subunits are found in a broad spectrum of human cancers; however, the mechanisms of how these aberrations of SWI/SNF complex would impact tumorigenesis and cancer therapeutics remain to be elucidated. Studies have demonstrated that immune checkpoint blockade (ICB) therapy is promising in cancer treatment. Nevertheless, suitable biomarkers that reliably predict the clinical response to ICB are still lacking. Emerging evidence has suggested that SWI/SNF components play novel roles in the regulation of anti-tumor immunity, and SWI/SNF deficiency can be therapeutically targeted by ICB. These findings manifest the prominence of the SWI/SNF complex as a stratification biomarker that predicts treatment (therapeutic) response to ICB. In this review, we summarize the recent advances in ICB therapy by harnessing the cancer-specific vulnerability elicited by SWI/SNF deficiency. We provide novel insights into a comprehensive understanding of the underlying mechanisms by which SWI/SNF functions as a modulator of anti-tumor immunity.

## Introduction

The mammalian switch/sucrose non-fermentable (SWI/SNF) family is a multi-subunit chromatin remodeling complex that utilizes the energy of ATP hydrolysis to remodel nucleosomes and regulates DNA accessibility in fundamental cellular processes, including transcription, DNA repair, and replication^1^. SWI/SNF complex mutations are frequently observed in a broad spectrum of human cancers^2–5^. There is increasing evidence regarding the critical biological functions of the SWI/SNF complex in cancer; however, the underlying mechanisms by which SWI/SNF components contribute to tumorigenesis or drug sensitivity warrant further investigation. Importantly, it remains unknown whether and how SWI/SNF mutations or defects could be exploited for therapeutic purposes^6^.

Cancer immunotherapy especially immune checkpoint blockade (ICB) has recently become one of the most prominent therapeutics for human cancers^7^. Briefly, the immune checkpoint constitutes a negative regulatory mechanism that maintains immune tolerance and prevents the overactivation of immune responses^8^. This mechanism can be hijacked by tumor cells to avoid elimination by immune cells, thus ICB therapy (e.g., anti-CTLA-4, anti-PD-1/PD-L1) was developed to counteract the immune suppressive microenvironment, thereby to strengthen the tumor-killing effects^9^. ICB therapy has been approved to treat multiple cancer types^10–18^. But the clinical efficacy of current ICB therapy is low; only a minority of patients may respond (<30% overall response rate)^19^. Furthermore, a considerable proportion of patients (25–30%) may develop immune-related adverse events or fatal outcomes^20^. In addition, up to two-thirds of ICB therapy recipients may exhibit either primary or acquired resistance^21–23^. These observations highlight the need for elucidation of resistance mechanisms, the proposal of more effective therapeutic strategies, and more importantly identification of effective biomarkers for stratifying cancer patients^24^.

While more effective than monotherapy, the combination ICB approaches are likely to exacerbate the incidence of immune-related adverse events that could greatly impact the clinical feasibility of combination therapy^25^. In this regard, the identification of biomarkers that predict patients who are more likely to respond to ICB therapy is of considerable importance. Recently, we and others have reported that SWI/SNF deficiency is related to sensitivity to immune checkpoint blockade (ICB) therapy, indicating the potential for use of the SWI/SNF complex as a target for cancer immunotherapy^6,26–29^. These findings suggest a novel role for SWI/SNF in modulating anti-tumor immunity and imply that aberrations of SWI/SNF components may serve as biomarkers to predict patient response to clinical ICB therapy. In addition, these results further support the notion that aberrations of SWI/SNF members can be therapeutically targeted^30–33^. The synthetic lethal effects and mechanisms of SWI/SNF subunits have been extensively reviewed elsewhere^34–36^. Here, we summarize the current understanding of mechanisms of molecular vulnerability mediated by SWI/SNF core members and the therapeutic applications in ICB.

## SWI/SNF complex: a highly mutated chromatin remodeler in human cancers

**Fig. 1:**
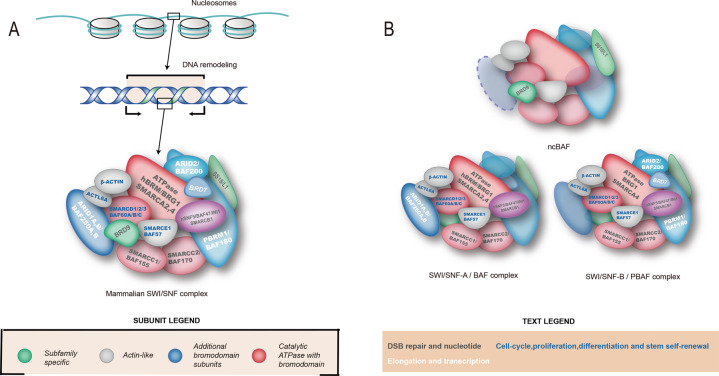
The structure and composition of the mammalian SWI/SNF complex. **A** The subunits of the SWI/SNF complexes, including catalytic ATPase with bromodomain, additional bromodomain subunits, and accessory subunit. Different colors of subunits and characters in the complex indicate the function of various subunits. **B** Three major complexes in mammalian SWI/SNF complex: BRG1-associated factor (BAF; also known as SWI/SNF-A) complexes; polybromo BRG1-associated factor (PBAF; also known as SWI/SNF-B) complexes; and noncanonical BAF (ncBAF/GBAF) complexes.

SWI/SNF complex consists of 15 subunits encoded by up to 29 genes and possessed ATP-dependent nucleosome remodeling activity (Fig. 1A)^37,38^. Based on the subunit composition, three major complexes in mammals: BRG1-associated factor (BAF; also known as SWI/SNF-A) complexes; polybromo BRG1-associated factor (PBAF; also known as SWI/SNF-B) complexes; and noncanonical BAF (ncBAF/GBAF) complexes. They exist in various compositions and proteins encoded by paralogous genes may alternately occupy several positions (Fig. 1B) (Table 1).

**Table 1:** Subunits of chromatin remodeling complexes: SWI/SNF and PRC1,2.

Subunits of chromatin remodeling complexes: SWI/SNF and PRC1,2	
mSWI/SNF	DOMAINS
*BRG, BRM*	ATPase, bromodomain, HSA, BRK
*BAF170/SMARCC2*	Chromo-related domain, SWIRM, SANT, Leu-zipper
*BAF155/SMARCC1*	Chromo-related domain, SWIRM, SANT, Leu-zipper
*BAF57*	HMG, coiled coil
*BAF47/hSNF5/INI/SMARCB1*	SNF5 domain
*BAF60A, B, C*	SWIB/MDM2 domain
β-actin	Actin
*ARID1A/BAF250A*	ARID
*ARID2/BAF200*	ARID, RFX, Zn finger
*PBRM1/BAF180*	Bromodomain, BAH, HMG
PRC1	
CBX2/HPC1, CBX4/HPC2, CBX6, CBX7, CBX8/HPC3	Chromodomain, AT-hook
PHC1,2, 3	FCS Zn finger, SAM domain
RING1A, RING1B	RING1A, RING1B RING finger
BMI1, MEL, Bmi1, Mel18, MBLR, NSPc1	RING finger
PRC2	
EZH1, EZH	SET, SANT
EED	WD40
SUZ1ZN	Zn finger

SWI/SNF complex mutations are frequently observed in numerous human cancers, with an average mutation rate of 20%, which ranks immediately after p53 (26%) (Table 2)^2–4^. By far, the most commonly mutated BAF subunit in cancer is *ARID1A* (BAF250A), the AT-rich interacting domain-containing protein 1A, which is mutated in 50% of ovarian clear-cell carcinoma (OCCC), 11% of colorectal adenocarcinoma^3,39–43^ and 9% of endometrial carcinoma^44^, 17.5% of colon and rectal cancers^45^, certain pancreatic cancer^46^, 13% of transitional cell carcinoma of the bladder^47^, 27% of gastric cancer^41,48^, 16.7% of cholangiocarcinoma^49^, and 11% of childhood neuroblastoma^50^. *BRG1/SMARCA4* is mutationally inactivated or epigenetically silenced in non-small-cell lung cancer (NSCLC), medulloblastoma, and Burkitt’s lymphoma^51–53^. In addition, ~90% of malignant rhabdoid tumors (MRT) harbor *SNF5*/*SMARCB1* (integrase interactor 1/SWI/SNF‐related matrix‐associated actin‐dependent regulator of chromatin subfamily B member 1) mutations^54^. The PBAF complex component *PBRM1 (BAF180)* contains six bromine domains and is mutated or deleted in >50% of clear-cell renal cell carcinoma (ccRCC)^55^. Overall, *ARID1A* is the most frequently mutated SWI/SNF subunit in different cancer types; however, *PBRM1* mutations are much more common in ccRCC than *ARID1A* mutations^6^. These mutations usually cause the effects of loss-of-function (LOF) to almost all the subunits; however, these incidences have shown a cancer-dependent fashion, indicating the function of the SWI/SNF complex is context-specific^56^. With mutations in the SWI/SNF subunits found in ~25% of cancers, the question of whether such mutations have therapeutic implications naturally arises.

**Table 2:** Mutated SWI/SNF components in human cancers.

SWI/SNF subunits	Cancer type/phenotypes
*BRG1/SMARCA4*	NSCLC, medulloblastoma, Burkitt’s lymphoma, lung cancer
*hSNF5/BAF47/INI1/SMARCB1*	Malignant rhabdoid tumor (MRT) and kidney malignancies
*ARID1A/BAF250A*	Endometrial carcinoma, colon and rectal cancers, pancreatic cancer, transitional cell carcinoma of the bladder, gastric cancer, cholangiocarcinoma, childhood neuroblastoma, and ovarian clear-cell carcinoma
*SMARCC1/BAF155*	Breast cancer progression and metastasis, colon cancer cell, pancreatic ductal adenocarcinoma, and melanoma
*PBRM1*	Clear-cell renal cell cancer (ccRCC)

## SWI/SNF core subunits as potential biomarkers of ICB therapy

The investigations of biological functions of the SWI/SNF complex are currently developing and the molecular consequences resulted from subsequent mutations associated with core complex subunits in human cancers are not yet fully understood^6^. Here, we focused on the core subunits of the SWI/SNF complex that could serve as functional biomarkers for ICB therapy.

### PBRM1

Studies in ccRCCs patients have shown that the loss of biallelic *PBRM1*, a PBAF component of the SWI/SNF complex, is positively associated with better response to anti-PD-1 or anti-PD-L1 treatment, regardless of mutation burden^57,58^. Mutations in *PBRM1*, *ARID2*, and other SWI/SNF components are also overrepresented in complete or partial responders, compared with non-responders^6,57,59^. Progressive ccRCC tumors exhibit high levels of CD8^+^ T-cell infiltration, compared with noninvasive tumors; notably, infiltrating tumors have reduced levels of favorable *PBRM1* mutations. The most commonly mutated genes (e.g., *VHL*, *PBRM1*, *SETD2*, *BAP1*, and *KDM5C*) were recurrent in both earlier and advanced disease^58^. However, the precise role of PBRM1 in the ccRCC immune microenvironment remains unclear, because contradictory results have shown that loss of PBRM1 reduced the binding of *BRG1* to the interferon (IFN)-γ receptor 2 promoters; STAT1 phosphorylation and subsequent expression of IFN-γ target genes, therefore correlated with a nonimmunogenic phenotype^60^.

### ARID1A

We have reported that *ARID1A* mutations positively correlate with enhanced anti-tumor immunity in both experimental models and patient samples, and ARID1A-mutant tumors are sensitized to anti-PD-L1 treatment^26^. Okamura et al. recently used next-generation sequencing technology to analyze 3403 patients receiving ICB therapy; they found >5% prevalence of *ARID1A* alterations in 1540 patients with nine distinct types of cancer^61^. The median progression-free survival of patients with altered *ARID1A* was significantly longer than that of patients with wild-type *ARID1A* in terms of response to ICB therapy, regardless of microsatellite instability (MSI) and tumor mutation burden (TMB) statuses. Because inhibition of histone deacetylase 6 suppresses the growth of *ARID1A*-mutated tumors, the combination of histone deacetylase 6 suppression and ICB represents a potential treatment strategy for *ARID1A*-mutated cancers^62^. More recently, Goswami S et al. showed that ARID1A depletion indeed correlated with improved sensitivity to anti-PD-1 therapy in a murine models of bladder cancer and in clinical cohorts. Importantly, biomarker combining *ARID1A* mutation and CXCL13 expression exhibits even better prediction power of patient responses to ICB^29^. These results indicate that *ARID1A* status may predict patient responses to ICB therapy. Of note, Li J et al. have reported contradictory findings that *ARID1A* mutations led to impaired chromatin accessibility to IFN-responsive genes, and represented a poor anti-tumor immunity^63^. Therefore, *ARID1A* alteration merits further exploration as a novel biomarker for outcomes after ICB therapy^64^.

### SMARCB1/SNF5

Inactivation of biallelic *SMARCB1/SNF5* is largely restricted to the rare pediatric rhabdoid tumors. The vast majority of the rhabdoid tumors (over 95%) harbor *SMARCB1* mutations, or rarely *SMARCA4* mutations (<5%)^65^. Experimental rhabdoid tumor models have demonstrated significant regression of established tumors under ICB therapy, up to 67% of the mice with the *SMARCB1*-mutant rhabdomyoma readily responded to anti-PD-1 treatment^66^. Though the underlying mechanisms are not fully understood, SMARCB1-dependent activation of interferon signaling was observed in the modulation of immunogenicity^66^. The genetic background of rhabdoid tumors and a significantly higher responding rate suggest that *SMARCB1*-mutant tumors can be therapeutically targeted by ICB therapy. However, further studies employing clinical cohorts are required to test the prediction ability of SMARCB1 status to ICB.

### SMARCA4/BRG1

SMARCA4 deficiency reportedly correlated with improved responses to ICB therapy. A 3-year follow-up study of 126 squamous cell carcinoma of the head and neck (SCCHN) patients receiving anti-PD-1/L1 therapy demonstrated that *SMARCA4* mutation and/or frameshift were more frequently observed in responders than non-responders^67^. Consistently, a case report of thoracic sarcoma showed that SMARCA4 deficiency resulted in notable clinical response to Nivolumab (anti-PD-1) treatment^68^. Of note, in a small cohort study, four small-cell carcinomas of the ovary, hypercalcemic type (SCCOHT) patients showed a notable response to anti-PD-1 immunotherapy. Given that SCCOHT is a *SMARCA4* mutation-driven, highly aggressive monogenic cancer type, it may reflect the enhanced immunogenicity mediated by loss of SMARCA4 function^69^. Emerging data indicate that alternative epigenetic enzymes, such as lysine-specific demethylase 1 (LSD1), could induce an anti-tumor immune response in the scenario of SWI/SNF inactivation. LSD1 is highly expressed in SWI/SNF-mutant SCCOHT tumors and the inhibition of LSD1 activity exhibits remarkable anti-tumor efficacy when combined with immune checkpoint blockade^70^. These findings suggest the potential of LSD1 as a target for the combinational immunotherapy of SWI/SNF-mutated tumors. However, these results require studies with a larger cohort of patients and longer follow-up to validate.

### ICB clinical trials targeting SWI/SNF-mutant tumors

Multiple immune checkpoint inhibitors (listed in Table 3) are currently under investigation to exploit aberrations of SWI/SNF components^71^: nivolumab, a fully human IgG4 PD-1 antibody; pembrolizumab (i.e., MK-3475 or lambrolizumab), a high-affinity humanized IgG4 monoclonal antibody targeting PD-1; and MPDL3280A, an engineered IgG anti-PD-L1 antibody. Notably, the feasibility of the SWI/SNF complex as a biomarker is still, to a certain extent, under debate. One recent cohort study showed that loss-of-function mutations in SWI/SNF components failed to predict improvements in overall survival, time to treatment failure, and disease control rate. However, patients harboring *PBRM1* mutations exhibited significantly improved overall survival and time to treatment failure^72^. These results further highlight the context-dependent functions of the SWI/SNF complex, which warrant large cohort and multicancer studies.

**Table 3:** Recent clinical trials of immune checkpoint blockades targeting the aberration of SWI/SNF subunits.

	When	Drug/combination	Aim	Cancer/stage	Target	Reference
I	2018	Nivolumab	Anti-PD-1 mAbs	Metastatic clear-cell renal cell carcinoma (ccRCC)	*PBRM1*	Miao et al.^57^
II	2018	Ipilimumab	Anti-CTLA-4 mAbs	Translocation renal cell carcinoma (tRCC)	*PBRM1, BRD8*	Boilève et al.^90^
III	2018	ENMD-2076	Anti-PD-1 mAbs	Ovarian clear-cell carcinoma (OCCC)	*ARID1A*	Lheureux et al.^91^
IV	2019	Gemcitabine	GP	Ovarian clear-cell carcinoma (OCCC)	*ARID1A*	Kuroda et al.^92^
V	2020	Toripalimab	Anti-PD-1 mAbs	Recurrent or metastatic neuroendocrine neoplasms (NENs)	*ARID1A*	Lu et al.^93^
VI	2018	M6620	Anti-PD-1 mAbs	Small-cell lung cancer (SCLC)	*ARID1A*	Thomas et al.^94^

## Mechanisms of SWI/SNF-mediated immune modulation

### Silencing of IFN-stimulated genes

Mutations in *PBRM1* are associated with the enrichment of gene expressions that stimulate immune responses (e.g., hypoxia responses and JAK-STAT signaling) in ccRCC^57^. In addition, inactivation of *PBRM1* sensitizes tumor cells to T-cell-mediated cytotoxicity and results in a more favorable tumor microenvironment^59^. An important role of *PBRM1* in immune modulation is the suppression of IFN-γ-responsive gene expression, thus conferring T-cell-mediated killing resistance to tumor cells. Pan et al. have found that inactivation of PBRM1 enhances the chromatin accessibility of transcription factors on the promoters of many IFN-γ-inducible genes, therefore leads to the suboptimal response to ICB therapies^59^. They also discovered that decreased interferon signaling can lead to lost opportunities for the upregulation of PD-L1, an established downstream target of IFN-γ^59^. Moreover, *PBRM1* functions as a synergistic factor with EZH2, which promotes its silencing effect upon IFN-stimulated genes; this suggests a possible mechanistic explanation for the positive relationship of PBAF loss with the reduction of IFN-stimulated gene expression^73^. Notably, Th1-type chemokines (e.g., CCL5, CXCL9, CXCL10, and CXCL11) are top-level genes that are differentially regulated by both EZH2 and *ARID1A*. Furthermore, the expression level of ARID1A was positively correlated with the levels of IFN-responsive genes^63^. EZH2 has been previously shown to suppress Th1-type chemokine (primarily CXCL9 and CXCL10) expression and alter effector T-cell tumor trafficking^74,75^. Therefore, *PBRM1* also downregulates the expression of innate immune-related chemokines^76^.

**Fig. 2:**
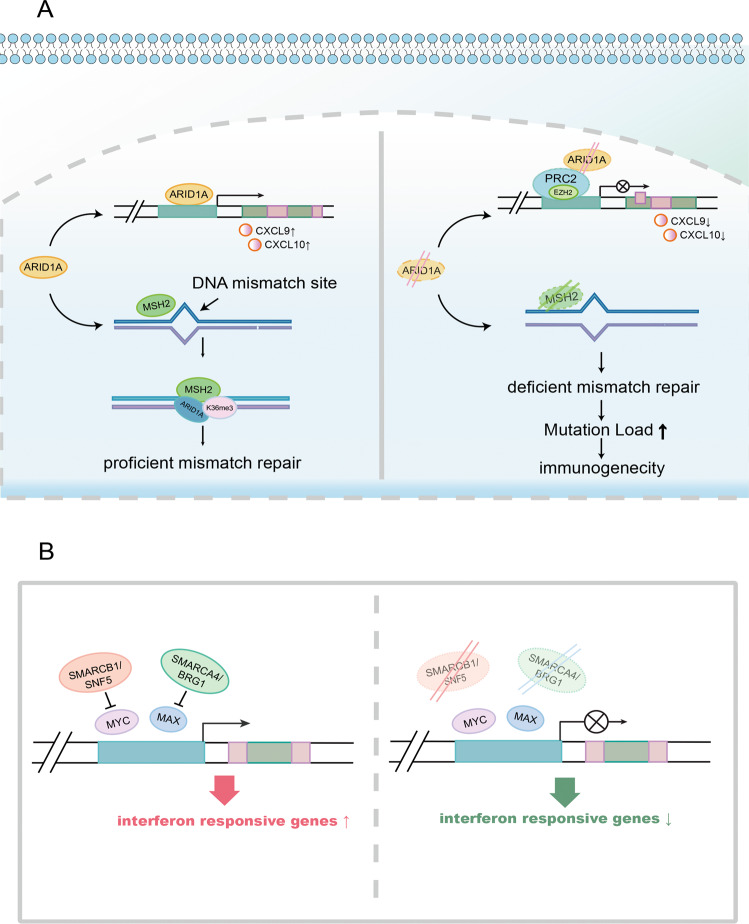
Mechanisms of SWI/SNF-mediated immune modulation. **A** ARID1A modulates DNA mismatch repair and the expression of IFN-responsive genes. ARID1A restricts the chromatin accessibility of EZH2 and PRC2 complex to IFN-responsive genes. The aberration of ARID1A attenuates IFN gene expression and poor Th1-type chemokine expression (e.g., CXCL9 and CXCL10). ARID1A interacts with MMR protein MSH2 and functionally regulates MSH2 positioning at DNA mismatch sites, tumor mutation load, and the subsequent immunogenicity. **B** SNF5 and BRG1 modulate the expression of IFN-responsive genes through MYC and MAX. SWI/SNF subunits SNF5 and BRG1 interact with MYC and MAX, respectively, therefore alleviate the inhibitory function of MYC and MAX upon IFN-responsive genes. SNF5 interacts directly with MYC, through MYC HLH-LZ and SNF5 Rpt motifs. BRG1 interacts and regulates MAX, a functional partner of MYC.

*ARID1A* aberrations also have been shown to restrict the accessibility of chromatin to IFN-responsive genes, resulting in attenuated IFN gene expression and poor Th1-type chemokine expression (Fig. 2B)^63^. Through a synergistic effect with phosphatidylinositol 3-kinase signaling, *ARID1A* inhibits inflammation-driven tumorigenesis by limiting the production of interleukin-6^77^. In addition, *ARID1A* interacts with EZH2, an enzymatic subunit of the Polycomb complex, through its carboxyl-terminal; this interaction antagonizes EZH2-mediated IFN reactivity^63^. Importantly, the loss of *ARID1A* may synergize with phosphatidylinositol 3-kinase/AKT signaling activation, further enhancing oncogenic signaling^36^.

### Interactions with MYC in immune regulation

SWI/SNF complexes also interact with MYC, a well-characterized transcription factor, and master regulator of anti-tumor immune responses^78–80^. The link between MYC and epigenetic regulation is the interaction of MYC and SNF5 (SMARCB1/INI/BAF47), a core member of the SWI/SNF chromatin remodeling complex^81^.

Liu et al. described the negative regulation of the MYC-mediated BRD7 subunit, which is consistent with our findings regarding the co-expression correlation analysis of BRD7^82^. The oncogene MYC is regarded as the central hub, which may regulate the expression of SWI/SNF subunits: (1) MYC protein interacts with the SWI/SNF subunit *BAF47/hSNF5*^83^; (2) MYC localizes the SWI/SNF complex to the target gene locus that will undergo transcriptional regulation^84^; (3) the SWI/SNF complex can regulate MYC through transcription. MYC and SNF5 interact directly, through important functional domains in both proteins, including MYC HLH-LZ and SNF5 Rpt motifs^85^. The interaction of SNF5 with MYC impedes the DNA-binding activity of MYC at certain target genes^83^. In addition, BRG1 is reported to regulate MAX, a functional partner of MYC, therefore alleviate the inhibitory function of MAX and/or MYC at the chromatin region of IFN-responsive genes^86^.

### Regulation of DNA mismatch repair

DNA mismatch repair (MMR) maintains replication fidelity by correcting mismatched nucleotides bound by DNA polymerases^87,88^. The loss of DNA mismatch repair activity leads to MSI, a hypermutable phenotype^89^. *ARID1A* has been reported to interact with MMR protein MSH2 and functionally regulate MSH2 positioning at DNA mismatch sites without affecting MSH2 expression. *ARID1A* deficiency impairs MMR efficiency and causes a mutator phenotype in both cancer cell lines and in vivo tumor samples (Fig. 2A). ARID1A deficiency is associated with the genomic features of a C > T mutation pattern and markedly elevated TMB, which are commonly observed in MMR-deficient samples. Importantly, *ARID1A*-deficient tumors are sensitive to anti-PD-L1 treatment in syngeneic mouse models^26^.

Clinical studies have shown that MSI and/or MMR deficiency sensitizes tumors to ICB therapy; therefore, MSI and MMR deficiency have received expedited the United States Food and Drug Administration approval for use as a patient stratification biomarker in the treatment of solid tumors, regardless of cancer types^17,18^. However, current methods for MSI determination are based on the detection of abnormalities at genomic loci or the loss of MMR proteins (e.g., MLH1); thus, functional defects in MMR may not be readily identified. For instance, trimethylation of the histone H3 lysine 36 recruits Mutsα to the replicating chromatin and facilitates MMR; defects in histone H3 lysine 36 activity result in functional impairment of MMR, rather than clinically validated MSI^87^. These results manifest the importance of functional biomarker of MMR (e.g., ARID1A status) to predict the responses to ICB therapy.

## Conclusions

The discovery that the SWI/SNF complex plays an essential role in determining the therapeutic efficacy of cancer immunotherapy highlights several important future goals. First, we note that controversial results were reported regarding the function of SWI/SNF aberration in predicting the clinical responses to ICB therapy (e.g., *ARID1A* and *PBRM1* alterations from different research groups), therefore further studies require longer follow-up durations and larger cohorts of patients to further determine the clinical feasibility. Second, most of the current findings are based on phenotypic observations that lack mechanistic insights. Thus, the underlying mechanisms by which SWI/SNF complex members could modulate anti-tumor immunity and/or responses to ICB therapy warrant further examination. It is crucial to determine how SWI/SNF complex-mediated chromatin remodeling could modify immune cell function in the tumor microenvironment, to elucidate whether these regulatory mechanisms of SWI/SNF could be exploited to refine the immune checkpoint networks and to identify the context-dependent binding partners of SWI/SNF that could be targeted to achieve durable ICB therapeutic effects. In addition, a single-target biomarker may lack enough prediction power. In this regard, a combinational biomarker that based on SWI/SNF complex and other markers (e.g., PD-L1, CXCL13) could be further investigated in the future.
